# Characterisation of Aerotolerant Forms of a Robust Chicken Colonizing *Campylobacter coli*

**DOI:** 10.3389/fmicb.2017.00513

**Published:** 2017-03-27

**Authors:** Peter M. O’Kane, Ian F. Connerton

**Affiliations:** Division of Food Sciences, School of Biosciences, University of NottinghamSutton Bonington, UK

**Keywords:** *Campylobacter coli*, aerotolerant *Campylobacter*, chicken intestinal colonization, oxidative stress, *Campylobacter* survival, food safety

## Abstract

*Campylobacter* contaminated poultry meat is a major source of human foodborne illness. *Campylobacter coli* strain OR12 is a robust colonizer of chickens that was previously shown to outcompete and displace other *Campylobacter* strains from the chicken’s gastrointestinal tract. This strain is capable of aerobic growth on blood agar. Serial aerobic passage increased this aerotolerance as assessed by quantitative assays for growth and survival on solid media. Aerotolerance was also associated with increased peroxide stress resistance. Aerobic passage did not alter cellular morphology or motility or hinder the microaerobic growth rate. Colonization of broiler chickens by aerotolerant *C. coli* OR12 was significantly lower than the wild-type strain at 3 days after challenge but not by 7 days, suggesting adaptation had occurred. Bacteria recovered from chickens had retained their aerotolerance, indicating this trait is stable. Whole genome sequencing enabled comparison with the wild-type sequence. Twenty-three point mutations were present, none of which were in genes known to affect oxidative stress resistance. Insertions or deletions caused frame shifts in several genes including, phosphoglycerate kinase and the b subunit of pyruvate carboxylase that suggest modification of central and carbohydrate metabolism in response to aerobic growth. Other genes affected include those encoding putative carbonic anhydrase, motility accessory factor, filamentous haemagglutinin, and aminoacyl dipeptidase proteins. Aerotolerance has the potential to affect environmental success and survival. Increased environmental survival outside of the host intestinal tract may allow opportunities for transmission between hosts. Resistance to oxidative stress may equate to increased virulence by virtue of reduced susceptibility to oxidative free radicals produced by host immune responses. Finally, resistance to ambient atmospheric oxygen may allow increased survival on chicken skin, and therefore constitutes an increased risk to public health.

## Introduction

*Campylobacter* spp. have been the most commonly reported gastrointestinal pathogens in the EU since 2005, with 229,213 confirmed cases in 2015, representing 65.5 per 100,000 of population (EFSA, 2016). England and Wales reported 65,032 laboratory confirmed cases of campylobacteriosis in 2012 (PHE, 2013). This is a significant underestimate due to under-diagnosis and under-reporting. A recent study estimated that in 2009 there were 280,400 cases of campylobacteriosis in the UK, accounting for 38,860 general practitioner consultations and 562 hospital admissions (O’Brien et al., 2016). The economic costs associated with *Campylobacter* infections over the same period was estimated to be £50 million (Tam and O’Brien, 2016). The majority of these cases are associated with *Campylobacter jejuni* infection but it is estimated that *C. coli* represents 7% of the cases in the UK (Gillespie et al., 2002).

The primary feature of disease is diarrhea, which can be accompanied by abdominal pain, fever, dysentery, vomiting, or prostration. Most cases are self-limiting with duration of illness varying from days to weeks. Illness can be more severe or life threatening in the young, old and immunocompromised (Blaser and Engberg, 2008). Of the 229,213 reported campylobacteriosis cases in the EU in 2015, 19,302 required hospitalization (31.2%), and there were 59 deaths (0.03%) (EFSA, 2016). In addition to the acute gastrointestinal disease, *Campylobacter* infection can be associated with a number of severe post-infectious complications. The most significant of these are believed to be Guillain-Barré syndrome, reactive arthritis, irritable bowel syndrome, and inflammatory bowel disease (WHO, 2013). Many other sequelae are described including: pancreatitis, septic abortion, cholecystitis, nephritis, peritonitis, and myocarditis (Blaser and Engberg, 2008).

Broiler meat is considered to be the main source of human campylobacteriosis (Wilson et al., 2008; Sheppard et al., 2009), with undercooking or cross contamination likely to be the main routes of infection (EFSA, 2016). Consumption of undercooked or contaminated poultry meat is the largest single risk factor for sporadic infection, and other sources are described such as milk, water, other meats, and contact with pet or farm animals (Jacobs-Reitsma et al., 2008). *Campylobacter* colonization is widespread throughout poultry flocks. A recent European Food Safety Authority baseline survey found 75.3% of UK broiler flocks to be positive for *Campylobacter* (EFSA, 2010). In 2014, 76% of 497 chicken neck skin samples collected from UK slaughterhouses tested positive for *Campylobacter* (EFSA, 2015). Caecal or crop contents of broilers at slaughter can contain up to 10^9^ or 10^5^ CFU/g, respectively, presenting a huge potential for the contamination of meat during evisceration (van Gerwe et al., 2010).

Resistance to oxidative stress is essential for *C. jejuni* colonization of the gastrointestinal tract (Hermans et al., 2011). Common sources of oxidative free radicals include the organisms’ own respiratory metabolism, the ambient atmosphere in the extra-intestinal environment, and reactive oxygen species (ROS) generated by phagocytic or cytotoxic cells involved in the host immune response (Atack and Kelly, 2009). ROS damage DNA, proteins and lipids, limiting growth or killing the bacteria (Flint et al., 2016a). *C. jejuni* lacks several of the key oxidative stress response regulators identified in *Escherichia coli*, such as SoxRS, OxyR or Crp, and whereas *E. coli* possesses three separate superoxide dismutase and two catalase enzymes, *C. jejuni* encodes only one of each (Parkhill et al., 2000; Imlay, 2013; Flint et al., 2016b).

The three canonical enzymes involved in oxidative stress resistance in *C. jejuni* are superoxide dismutase, catalase, and alkyl hydroperoxide reductase. A superoxide dismutase (*sodB*) deficient mutant of *C. coli* had diminished environmental survival and decreased ability to colonize day old chicks (Purdy et al., 1999). A catalase (*katA*) deletion mutant *C. jejuni* demonstrated attenuated colonization fitness in a neonatal piglet model (Flint et al., 2012); however, a *katA* deficient *C. coli* had the same chick colonization characteristics as its parental strain (Purdy et al., 1999). Deletion of alkyl hydroperoxide reductase (*ahpC*) in *C. jejuni* significantly reduced its ability to colonize day old chicks (Palyada et al., 2009). Other peroxiredoxins involved in resistance to oxidative stress are the thiol peroxidase Tpx and bacterioferritin comigratory protein BCP (Atack et al., 2008). Methionine sulfoxide reductase enzymes MsrA and MsrB protect *C. jejuni* from R- and S-methionine sulfoxides, which are produced from the oxidation of methionine. Deletion of *msrA* and *msrB* from *C. jejuni* resulted in increased sensitivity to ROS assays (Atack and Kelly, 2008). The DNA–binding protein from starved cells (Dps) has also been identified as a colonization factor for *C. jejuni* in a chick model and has been studied as a potential antigen candidate for a subunit anti-*Campylobacter* vaccine (Theoret et al., 2012). Other proteins involved in oxidative stress resistance of *Campylobacter* include cytochrome c peroxidases, quinone reductases, and DNA repair proteins (Atack and Kelly, 2009; Flint et al., 2016b). The response to oxidative stress is coordinated by a complex and overlapping network of regulators. These include the peroxide regulator (PerR) and ferric uptake regulator (Fur) (Palyada et al., 2009), the *Campylobacter* oxidative stress regulator (CosR) (Hwang et al., 2011), and the regulators of response to peroxide (RrpA and RrpB) (Gundogdu et al., 2015).

*Campylobacter jejuni* and *C. coli* are considered to be obligate microaerophilic bacteria, requiring oxygen concentrations between 2 and 10% for optimal growth (Kaakoush et al., 2007). The primary stress encountered in the extra-intestinal environment may be the ambient 21% oxygen (Atack and Kelly, 2009). Reduced sensitivity to ambient oxygen would confer superior environmental resistance, therefore increasing the likelihood of transmission between potential hosts. Improved survival of particular strains on chicken carcasses would be of obvious public health importance as the risks they pose would be higher than previously anticipated. In addition, if aerotolerance is related to oxidative stress resistance, this may render the organism less sensitive to free radicals produced by the host’s immune response (Atack and Kelly, 2009), and therefore more pathogenic.

*Campylobacter coli* OR12 has been shown to be a highly successful colonizer of broiler chickens on organic and free-range farms (El-Shibiny et al., 2005). This strain is also capable of displacing other *Campylobacter* strains from pre-colonized chickens (El-Shibiny et al., 2007). An unusual characteristic of this strain is aerobic growth. Here we report adaptation of the strain to aerotolerance with the objective of determining whether the ability to withstand oxidative stress will modify the characteristics of *C. coli* OR 12 that make it a successful colonizer of broiler chickens.

## Materials and Methods

### Bacterial Strains

The *Campylobacter* strains used for this study were the chicken isolates *C. coli* OR12 (El-Shibiny et al., 2005), *C. coli* RM2228 (Fouts et al., 2005), *C. jejuni* HPC5 (Loc Carrillo et al., 2005), and the human clinical isolate *C. jejuni* NCTC 11168 (Parkhill et al., 2000).

### Aerobic Passage on Blood Agar (BA)

Aerotolerant *C. coli* OR12 (Aer) was serially passaged by streaking on blood agar plates (BA; Oxoid) containing 5% v/v defibrinated horse blood (TCS; Buckingham; UK) and incubating aerobically at 37°C for 2–7 days. Intermediate stocks were archived at regular intervals by collecting confluent aerobic growth from a BA plate into freezer stock solution (15% Glycerol, 85% Nutrient Broth No.2; Oxoid) and freezing at −80°C. Gram stains, using standard techniques, were performed at regular intervals to ensure contamination had not occurred. Four colonies were initially selected from the original growth and passaged independently. With the exception of pulsed-field gel electrophoresis (PFGE), all experiments were conducted using isolate B, as it had qualitatively demonstrated the best growth in the early passages. The original wild type *C. coli* OR12 (WT) was maintained and stored microaerobically on BA.

### Quantitative Aerobic Growth or Survival on BA

Blood agar 48 well plates were prepared by dispensing 1 ml of BA to each well (Nunclon Delta; Nunc; Denmark). Inoculum suspensions of approximately 10^7^ CFU/ml of microaerobically grown *C. coli* RM2228, *C. coli* OR12 WT*, C. coli* OR12 Aer and aerobically grown *C. coli* OR12 Aer were prepared by suspending confluent growth from BA plates into PBS. Triplicate wells, representing biological replicates, were inoculated with 10 μl of suspension (approximately 10^5^ CFU) per strain. Once dried, plates were incubated aerobically at 37°C. After 0, 6, 24, 30, and 48 h of incubation, well contents were suspended by gentle repeated pipetting in maximum recovery diluent (MRD; Oxoid). Suspensions were enumerated on CCDA using a modification of the Miles Misra method (Miles et al., 1938). Briefly, decimal dilutions were performed in MRD, 5 replicate 10 μl droplets from up to six dilutions were plated on modified *Campylobacter* blood-free selective agar (CCDA; Oxoid) with 2% agar. Once dry, plates were inverted, incubated microaerobically (5% CO_2_, 5% O_2_, 2% H_2_, 88% N_2_) in a modular atmosphere controlled cabinet at 42°C (Don Whitley Scientific modified atmospheric cabinet, Shipley, UK) and examined after 24 and 48 h. Dilutions giving rise to between 3 and 30 colonies were selected, the sum of the 5 spots was multiplied by 20, then multiplied by the inverse of the dilution factor to give the original CFU/ml.

### Qualitative Aerobic Growth on Solid Media (BA and CCDA)

Suspensions of approximately 10^8^ CFU/ml of the following strains were prepared: microaerobically grown *C. jejuni* NCTC 11168; *C. jejuni* HPC5; *C. coli* RM2228; *C. coli* OR12 WT; *C. coli* OR12 Aer and aerobically grown *C. coli* OR12 Aer. Each inoculum was decimally diluted five times and one 10 μl droplet of each dilution of each strain was dispensed onto two BA plates and two CCDA plates. Once dry, one of each was incubated microaerobically at 42°C and the other at 37°C aerobically. The microaerobic plates were examined after 48 h and the aerobic plates checked daily for 6 days.

### Survival in Aerobic Liquid Medium

Sterile 250 ml flasks containing 50 ml Mueller-Hinton broth (MHB; Oxoid) were inoculated in triplicate to an approximate cell density of 10^6^ CFU/ml of microaerobically grown *C. jejuni* HPC5 and *C. coli* OR12 WT and aerobically grown *C. coli* OR12 Aer P25. These were incubated aerobically at 37°C with orbital shaking at 100 rpm. Samples were collected at 0, 2, 4, 6, 24, and 30 h and *Campylobacter* enumerated on CCDA as above.

### Growth Rate in Microaerobic Nutrient Broth No. 2

Sterile 100 ml flasks containing 30 ml nutrient broth no.2 (NB2; Oxoid) were inoculated in triplicate with approximately 10^4^ CFU/ml of microaerobically grown *C. coli* RM2228 and *C. coli* OR12 WT and aerobically grown *C. coli* OR12 Aer P38. The flasks were incubated microaerobically in anaerobic gas jars using gas replacement (7.3% CO_2_, 5.6% O_2_, 3.6% H_2_, 83% N_2_) at 42°C with orbital shaking at 100 rpm (John et al., 2011). Samples were collected and *Campylobacter* enumerated on CCDA at 0, 2, 5, and 8 h.

### Motility Assays

Motility was assessed using a motility agar assay and via membrane filtration. For the agar assay, inocula were prepared from freshly grown aerobic cultures of *C. coli* OR12 Aer or microaerobic cultures of *C. coli* OR12 WT. A pipette tip was used to collect a small plug of colony growth, which was then stab-inoculated into the center of a motility agar (Mueller Hinton broth with 0.4% v/v agar) plate, without touching the bottom. Plates were incubated microaerobically at 42°C and examined after 48 h.

For the membrane filtration assays, sterile nitrocellulose membrane filters with 0.45 μm pore size and 47 mm diameter (Millipore) were aseptically placed in the center of BA plates. The centers of the filters were inoculated with 50 μl of *C. coli* suspension taking care not to contaminate the surrounding agar. Once the liquid had passed through the filters, they were aseptically removed with sterile forceps. Plates were inoculated in duplicate, with one incubated microaerobically at 42°C and one aerobically at 37°C.

### Peroxide Stress Assay

Resistance to peroxide stress was performed using a method similar to that of Rodrigues et al. (2015). Glass universal containers, filled with 10 ml of MRD containing 0, 0.5 or 1 mM H_2_O_2_ (Sigma; UK), were inoculated with identical optical densities of approximately 10^8^ CFU/ml microaerobically grown *C. coli* RM2228*, C. coli* OR12 WT*, C. coli* OR12 Aer P40 and aerobically grown *C. coli* OR12 Aer P40. Containers were incubated microaerobically at 42°C for 1 h with shaking at 100 rpm followed by immediate enumeration of *Campylobacter* on CCDA as above. Three universals were inoculated per strain, representing independent biological replicates.

### Transmission Electron Microscopy

Confluent BA cultures of microaerobically grown *C. coli* OR12 WT and aerobically grown *C. coli* OR12 Aer P37 were fixed in electron microscopy fixative (3% glutaraldehyde in 0.1 M cacodylate buffer) prior to centrifugation and resuspension in sterile water. Transmission electron microscopy (TEM) was performed at the Advanced Microscopy Unit, University of Nottingham. Samples were adsorbed onto copper Formvar/Carbon grids (AGS162-3; Agar Scientific) and negatively stained with 3% uranyl acetate. Grids were imaged using a Tecnai G12 biotwin TEM, run at 100Kv, with a SIS megaview camera system and Gatan Microscopy Suite software (Gatan Inc.).

### Pulsed-Field Gel Electrophoresis

Cell suspensions of four *C. coli* OR12 and aerotolerant derivatives were incorporated into agarose plugs, lysed and washed as described by Ribot et al. (2001). DNA plugs were cut into 2-mm slices and placed into 100 μl of 1x SuRE/Cut Buffer A (Roche) and equilibrated at 25°C for 15 min. This was replaced with 100 μl of 0.2 U/μl *Sma* I (Roche) in the same buffer and incubated for 2 h at 25°C. The plugs were incorporated in a gel consisting of 1% w/v Agarose (Bio-Rad) in TAE buffer. A 50–1000 kb DNA ladder (Lambda PFGE Ladder; New England BioLabs) was added as a marker. Electrophoresis was performed using a Bio-Rad CHEF-DRII. The gel was stained in 50 μg/ml ethidium bromide in TAE buffer and visualized under UV light using the Gel Doc XR system with the Quantity One basic software, version 4.6.5 (Bio-Rad).

### Chicken Colonization Assay

Ross 308 broiler chickens (*n* = 30) were obtained as day old chicks from a commercial hatchery (PD Hook; UK) and reared in biosecure conditions. Chicks were group reared in pens with a bedding of wood shavings and transferred to individual cages with an astroturf floor and environmental enrichment at 18 days of age. A 12-h light-dark cycle was followed for the duration of rearing. Temperatures were as outlined in the Code of Practice for the Housing and Care of Animals Bred, Supplied or Used for Scientific Purposes, with minor adjustments made as guided by bird thermoregulatory behavior. They received commercial broiler diets (starter, grower, and finisher) for the duration. Cloacal swabs were taken at day 18 and tested for *Campylobacter* by direct plating on CCDA and *Salmonella* by enrichment. At 21 days of age, 15 birds (Group 1) were gavaged with an estimated 10^8^ CFU of *C. coli* OR12 WT and 15 birds (Group 2) with *C. coli* OR12 Aer P34. After 3 days, seven birds from each group were culled by parenteral barbiturate overdose, with the remaining eight birds culled at 7 days post challenge. Caecal contents were collected and enumerated for *Campylobacter* in triplicate as described above, with CCDA selective supplement (Oxoid) added to the medium. The membrane filtration assay was also performed on the 10^−1^ dilution of each caecal content sample, as described above. A secondary aerotolerance assay was performed on 24 or 48 h growth on the microaerobically incubated BA filtration plates. Growth was suspended in PBS and normalized to an OD_600_ of 0.05, decimally diluted to 10^−3^ and a 10 μl spot of each dilution inoculated to a BA plate which was incubated aerobically at 37°C.

Power calculations were performed using the Spreadsheet for Sample Size Calculation (Brown, 2016). This indicated that seven birds were required per group to detect a 1 log_10_ CFU/g difference between groups with 95% confidence and a standard deviation of 0.5 log_10_ CFU/g. One additional bird was added per group to allow for any unexpected mortality.

### Ethics Statement

All experimental animal work was performed in accordance with UK and EU law. This study was approved by the Local Ethics Committee of the University of Nottingham and performed under Home Office license.

### Statistical Treatment of Data

Viable counts were log_10_-transformed for analysis. Statistically significant differences (*p* < 0.05) were assessed using parametric (ANOVA) and non-parametric (Mann–Whitney *U*) tests through the statistical packages available within Minitab statistical software (Minitab Inc.).

### Whole Genome Sequencing and Analysis

Genomic DNA was extracted from half of a confluent aerobically incubated BA plate of *C. coli* OR12 Aer P43. DNA was isolated using the GenElute Bacterial Genomic DNA Kit (Sigma–Aldrich) according to manufacturer’s instructions. Sequencing was performed using the MiSeq platform at Northumbria University. The sequence reads were aligned to the assembled sequence of *C. coli* OR12 (NCBI RefSeq NZ_CP013733.1) and appear in the database as CcOR12aero (NCBI RefSeq NZ_CP019977). DNA sequence variants were detected using the variant detector within CLC Genomics workbench 9.01 (Qiagen Bioinformatics). Candidate high probability nucleotide sequence variants detected were confirmed by careful inspection of the sequence reads, and any predicted changes to the protein encoding sequences confirmed using the Artemis genome browser (Rutherford et al., 2000). The predicted protein sequences were further compared with wild-type *C. coli* OR12 sequences, and with other similar protein sequences using the protein basic local alignment tool, BLASTP mounted on the NCBI website^1^. Nucleotide BLAST^1^ was used to identify orthologs of genes with known roles in *Campylobacter* oxidative stress resistance. Protein sequences of gene products in *C. jejuni* NCTC11168 and *C. coli* OR12 were compared using the protein BLAST function.

## Results

### Growth and Survival Characteristics of Aerotolerant *C. coli* OR12

**FIGURE 1:**
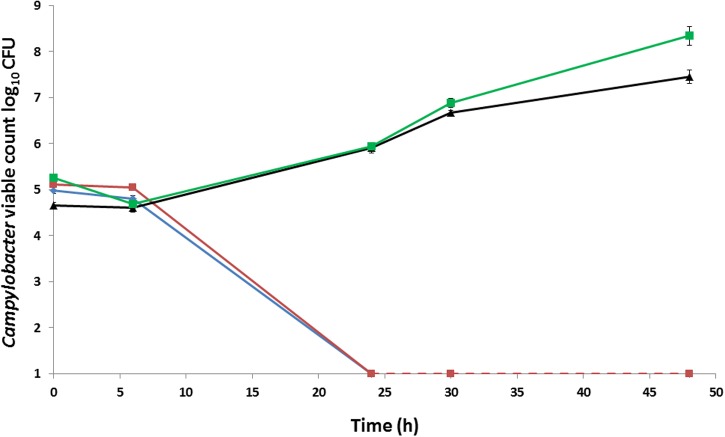
**Growth and survival of *Campylobacter coli* strains incubated aerobically on BA at 37°C.** *C. coli* RM2228 (blue), *C. coli* OR12 WT (red), aerobically prepared *C. coli* OR12 Aer P32 (black), microaerobically prepared *C. coli* OR12 Aer P32 (green). Error bars represent standard deviations from three biological replicates. Results are representative of three separate experiments. The limit of detection was 1.3 log_10_ CFU.

For the initial early passages, growth of aerotolerant *C. coli* OR12 was limited to the heavily inoculated region of the BA plate. After approximately 15 aerobic passages, growth of single colonies became visible within 2–5 days of aerobic incubation at 37°C. Gram staining confirmed typical *Campylobacter* morphologies with variable populations of Gram-negative spirals, filamentous, and coccoid forms. Approximately 10^5^ CFU of *C. coli* RM2228, wild type *C. coli* O12 (WT), aerotolerant *C. coli* OR12 (Aer) passage (P) 32, and a microaerobic preparation of *C. coli* OR12 Aer P32 were inoculated onto BA microplates and aerobically incubated at 37°C. No changes in viable count were apparent after 6 h; however, by 24 h the *C. coli* RM2228 and *C. coli* OR12 had declined to below the limit of detection (1.3 log_10_ CFU). In contrast, both the *C. coli* OR12 Aer P32 and the microaerobically prepared *C. coli* OR12 Aer P32 had grown by more than 1 log_10_ after 24 h and continued to grow, with viable counts of 7.4 and 8.3 log_10_ CFU, respectively, by 48 h (**Figure 1**).

**FIGURE 2:**
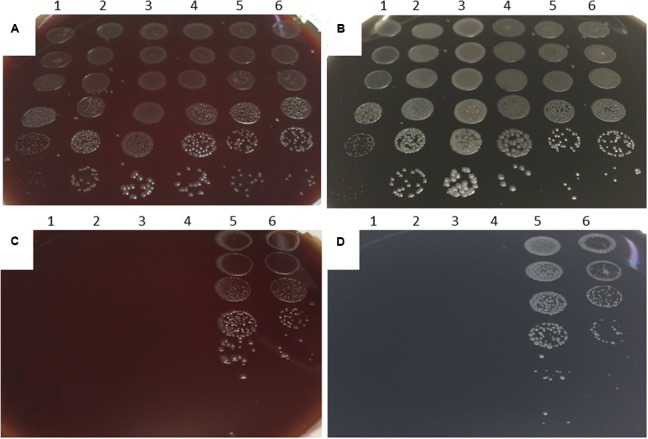
**Microaerobic and aerobic growth of *Campylobacter* on BA and CCDA.** Decimal dilutions of suspensions of: (1) *C. jejuni* NCTC 11168; (2) *C. jejuni* HPC5; (3) *C. coli* RM2228; (4) *C. coli* OR12 WT; (5) *C. coli* OR12 Aer P38 (aerobic prep); (6) *C. coli* OR12 Aer P38 (microaerobic prep) inoculated onto: Microaerobically incubated BA **(A)** and CCDA **(B)** after 48 h. Aerobically incubated BA **(C)** and CCDA **(D)** after 6 days.

Differences between aerobic and microaerobic growth were examined on BA and CCDA plates that had been inoculated with six decimal dilutions of a panel of six *Campylobacter* strains. These included *C. coli* OR12 WT along with aerobically and microaerobically prepared *C. coli* OR12 Aer P38. After 48 h of microaerobic incubation, single colonies were present at the highest dilution (10^−5^) of all strains on BA and CCDA, correlating with viable count ranges of between 5.7 × 10^7^ and 2.5 × 10^8^ CFU/ml for the initial inocula. Viable counts of all strains were within 1 log_10_ of each other (**Figure 2**). After 6 days of aerobic incubation, single colonies were present down to the 10^−4^ dilution of both aerobically and microaerobically prepared *C. coli* Aer P38 on BA and CCDA. No aerobic growth was present from any of the other strains examined (**Figure 2**). The growth rates of *C. coli* RM2228, *C. coli* OR12 WT and *C. coli* O12 Aer P38 were assessed by microaerobic incubation at 42°C in nutrient broth no. 2. Viable counts are shown in Supplementary Figure 1. Exponential growth rates for each strain were calculated in the logarithmic growth phase; rates were not significantly different between strains.

Aerobic survival in liquid medium was assessed by inoculating Mueller Hinton broth with an estimated 6 log_10_ CFU/ml and incubating at 37°C. Viable counts are presented in Supplementary Figure 2. Following 6 h of aerobic incubation, the *C. jejuni* HPC5, *C. coli* OR12 WT and *C. coli* OR12 Aer had declined by 0.17, 0.23, and 0.42 log_10_ CFU/ml, respectively. By 24 h, both *C. coli* OR12 strains and two of the *C. jejuni* HPC5 flasks had declined to below the limit of detection (1.3 log_10_ CFU/ml) and one *C. jejuni* HPC5 flask had a viable count of 2.4 log_10_ CFU/ml. Despite differences in the starting inocula, aerotolerant *C. coli* OR12 did not demonstrate superior survival compared to the wild type and *C. jejuni* HPC5.

### Aerotolerant *C. coli* OR12 Remain Motile in Atmospheric Oxygen

Motility assays were performed to determine whether serial aerobic passage resulted in reduced motility. The *C. coli* OR12 WT strain was tested together with *C. coli* OR12 Aer P22 and P50. All isolates demonstrated motility, with diameters of growth of 35, 34 and 33 mm, respectively, therefore aerotolerance was not associated with any loss of motility.

Owing to their highly energetic corkscrew-like motility campylobacters can traverse membrane filters that would exclude other bacteria, a property that has been used as a basis for their isolation from several animal sources (Moreno et al., 1993). Filtration was therefore used to examine the motility of aerotolerant *C. coli* OR12. Duplicate BA plates with centered nitrocellulose membrane filters were inoculated with 50 μl of suspensions containing 7.6 log_10_ CFU/ml *C. coli* OR12 WT or 7.6 log_10_ CFU/ml *C. coli* OR12 Aer P38. One of each was incubated microaerobically at 42°C and one of each aerobically at 37°C. After 24 h, heavy growth was present in the center of the filtered regions of both plates incubated microaerobically. After 4 days, growth was evident on the aerotolerant *C. coli* OR12 incubated aerobically, with confluent colonies by 5 days. No growth was noted on the wild type *C. coli* OR12 incubated aerobically. Under aerobic conditions aerotolerant *C. coli* OR12 remained motile and metabolically active to be able to translocate the membrane filter.

### Aerotolerant *C. coli* OR12 Demonstrates Increased Peroxide Stress Resistance

**FIGURE 3:**
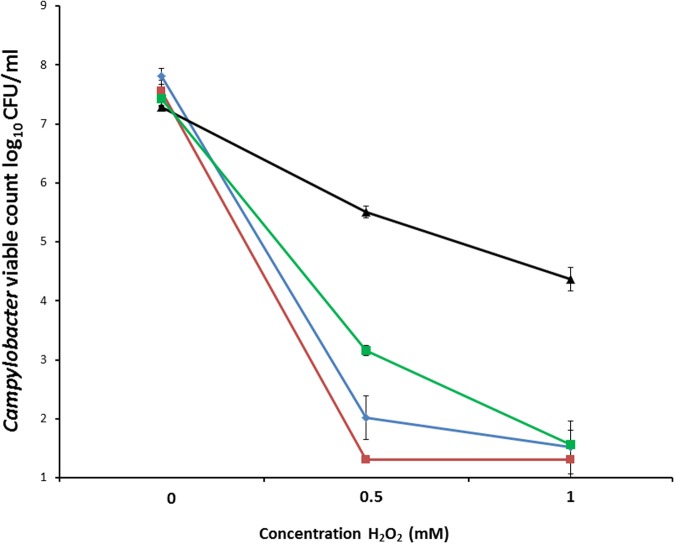
**Survival of *C. coli* in differing concentrations of hydrogen peroxide.** *C. coli* RM2228 (blue), *C. coli* OR12 WT (red), aerobically prepared *C. coli* OR12 Aer P40 (black), microaerobically prepared *C. coli* OR12 Aer (green). Cultures were incubated for 1 h at 42°C under microaerobic conditions. Error bars represent standard deviations from three biological replicates.

To measure oxidative stress resistance, suspensions containing between 7.3 log_10_ and 7.8 log_10_ CFU/ml *C. coli* RM2228, *C. coli* OR12 WT, aerobically prepared *C. coli* OR12 Aer P40 and microaerobically prepared *C. coli* OR12 Aer P40 were exposed to different concentrations of H_2_O_2_. Marked differences in survival were apparent between aerobically prepared aerotolerant *C. coli* OR12 and the other microaerobically prepared strains (**Figure 3**).

### Transmission Electron Microscopy

**FIGURE 4:**
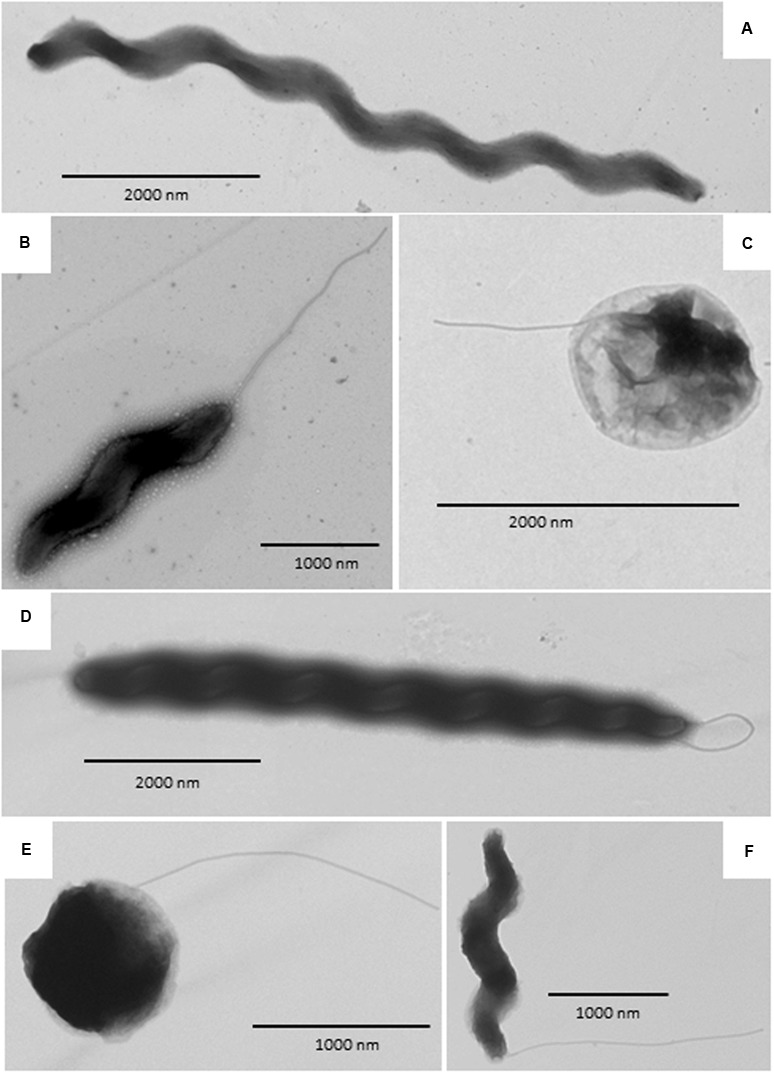
**Transmission electron microscopy images of *C. coli* OR12. (A–C)** Rrepresent wild type and **(D–F)** aerotolerant *C. coli* OR12. **(A)** Filamentous form; **(B)** Spiral form with flagellum at the poles; **(C)** Coccoid form with flagellum; **(D)** Filamentous form with flagella; **(E)** Coccoid form with flagellum; **(F)** Spiral form with single polar flagellum.

Transmission electron micrograph (TEM) images are shown in **Figure 4**. Both wild type and aerotolerant *C. coli* OR12 contained populations of typical spiral cells as well as filamentous and coccoid forms.

### Colonization of Chickens

**FIGURE 5:**
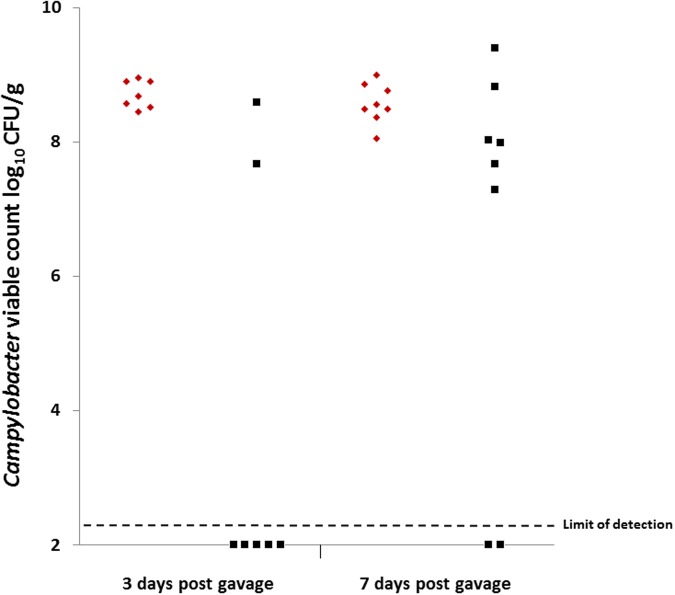
**Caecal *Campylobacter* counts from chickens challenged with, *C. coli* OR12 WT and Aer P34 at 3 and 7 days post challenge.** *C. coli* WT (red diamonds), *C. coli* OR12 Aer (black squares). Counts between wild type and aerotolerant strains were statistically significant 3 days post challenge (*p* = 0.006) but not at 7 days (*p* = 0.0659) using Mann–Whitney *U* tests.

Colonization gavage doses were titrated at 1.38 × 10^8^ CFU for group 1 (*C. coli* OR12 WT) and 3.4 × 10^7^ CFU for group 2 (*C. coli* OR12 Aer P34). Caecal *Campylobacter* counts at 3 and 7 days post gavage are shown in **Figure 5**. All the birds that received *C. coli* OR12 WT became colonized, with mean caecal *Campylobacter* counts of 8.71 (±0.2) and 8.57 (±0.3) log_10_ CFU/g at 3 and 7 days post gavage, respectively. Of the birds challenged with *C. coli* OR12 Aer, only 2/7 had detectably colonized by 3 days and 6/8 by 8 days. Birds with no *Campylobacter* growth were considered ≤2.3 log_10_ CFU/g, which was the limit of detection. The difference in caecal *Campylobacter* counts between the wild type and aerotolerant strain was significant at 3 days post challenge (*p* = 0.006), but not significant by day 7 (*p* = 0.0659) as determined by the two-tailed Mann–Whitney *U* test. Standard deviations for individual bird *Campylobacter* counts generated by triplicate technical replicates were all below 0.19 log_10_ CFU/g.

All birds from which *Campylobacter* was detected on CCDA also yielded positive growth on microaerobically incubated membrane filtered BA plates. Colony morphology was consistent with *Campylobacter* (Supplementary Figure 3) and Gram stains provided confirmation. Of the aerobically incubated membrane filtration BA plates, growth was detected from one of the *C. coli* OR12 Aer challenged birds at 3 days post challenge and from 3 birds at 7 days post challenge. No growth was detected on any of the aerobically incubated membrane filtered BA plates from wild type challenged birds.

Secondary aerotolerance assays were performed on the campylobacters isolated by microaerobic membrane filtration. All *C. coli* isolated from birds challenged with *C. coli* OR12 Aer P34 retained their aerotolerance. Supplementary Figure 3A shows the serially diluted and aerobically incubated *C. coli* isolated from the two birds 3 days post challenge with *C. coli* OR12 Aer P34. No such growth was noted from isolates recovered from the seven birds challenged with the wild-type strain.

### Whole Genome Sequencing and Analysis

Macrorestriction patterns of wild type *C. coli* OR12 and 4 aerobically passaged isolates determined by PFGE were identical to each other (Supplementary Figure 4) and to the original isolate described by El-Shibiny (2006). The development of aerotolerance is not associated with any significant DNA insertion, deletion or genome rearrangement. Therefore whole genome sequencing was performed to examine the occurrence of any point mutations.

A total of 23 mutations were noted between the wild type and aerotolerant *C. coli* OR12 sequences. There were 11 single nucleotide substitutions, 2 nucleotide insertions and 10 insertions or deletions within homopolymeric GC tracts. The DNA changes within homopolymeric GC tracts are summarized in **Table 1** and all others in **Table 2**. Three of the mutations were silent and one was within a non-coding region. All the predicted protein sequences were analyzed with BLASTP to confirm the protein identity and to determine if the changes induced were present in other similar sequences.

**Table 1:** Mutations in aerotolerant *Campylobacter coli* OR12 present within homopolymeric tracts.

Position	Consensus nucleotide change	Gene/CDS	Product	Change	AA change
455044	C (−)	ATE51_00892	PseD protein/Motility accessory factor	Premature termination	Y5 FS
455044	C (−)	ATE51_00894	Hypothetical protein/Motility accessory factor	Fusion to ATE51_00892	V60 FS
565851	G (−)	*hxuA*	Heme/hemopexin-binding protein precursor/filamentous hemagglutinin	Premature termination	M19 FS
828036	CC (+)	*pepD*	Aminoacyl-histidine dipeptidase	Premature termination	I186 FS
483767	G (−)	ATE51_00954	Hypothetical protein/carbonic anhydrase (incomplete)	C-terminal change	G196 FS
484990	G (−)	ATE51_00958	Hypothetical protein/carbonic anhydrase (incomplete)	Premature termination	S197 FS
1158035	CC (−)	ATE51_02354	Hypothetical protein/carbonic anhydrase	C-terminal change	G189 FS
64160	CC (−)	ATE51_00096	Iron-binding protein	C-terminal DR deletion	D240 FS
465041	CC (−)	ATE51_00914	Methyltransferase	C-terminal VL→VKL	V88 FS
366720	GG (−)	*yjjG*	Pyrimidine 5′nucleotidase/uridine kinase (incomplete)	C-terminal V→IG	V194 FS
422439	GG (−)	Non-coding	N/A	N/A	N/A

*Consensus nucleotide changes represent differences in the aerotolerant *C. coli* genome sequence by addition (+) or deletion (−) compared to the wild type sequence. FS indicates the change results in a frame shift.*

**Table 2:** Mutations in aerotolerant *C. coli* OR12 not within homopolymeric tracts.

Position	Nucleotide change	Gene/CDS	Product	Change	AA change
380879	A+	*pgk*	Phosphoglycerate kinase	Frame shift and premature termination	V19 frameshift
837990	T+	*cfiA*	Biotin attachment protein/Pyruvate carboxylase B subunit	Frame shift and premature termination	D419 frameshift
116044	C→T	*nhaA1*	Na/H antiporter	Single aa substitution	A166→V
358671	C→T	*epsE*	Glycosyl transferase family A	Single aa substitution	T367→I
615756	A→G	*mnmG*	tRNA uridine 5-carboxymethylaminomethyl modification enzyme GidA	Single aa substitution	T368→A
56663	G→A	*TypA/BipA*	GTP- binding protein	Single aa substitution	T210→I
168195	C→T	*sat*	Sulfate adenylyltransferase	Single aa substitution	A290→V
1346838	A→G	ATE51_02794	Hypothetical protein/VgrG type VI secretion protein (incomplete)	Single aa substitution	D19→G
1722359	G→A	*panC*	Pantoate beta alanine ligase	Single aa substitution	E216→K
1948793	C→T	*infB*	Translation initiation factor IF-2	Single aa substitution	R720→H
615323	C→T	*mnmG*	5-carboxymethylaminomethyl modification protein	Silent	None
1205648	T→C	*xerD*	Recombinase	Silent	None
1205660	A→G	*xerD*	Recombinase	Silent	None

### Phosphoglycerate Kinase

Gene *pgk*, which encodes the 400 amino acid protein phosphyglycerate kinase is located between nucleotides 380840–382042. BLASTP analysis of the amino acid sequence identified many *C. coli* and *C. jejuni* sequences with 99–100% coverage and identity. Aerotolerant *C. coli* OR12 has an A insertion at nucleotide position 380,879, which results in a frame shift at residue 19 and early protein termination at 21 amino acids. The entire amino acid sequence of the mutant protein is: MSDIISIKDIDLSKKKSFYKM. BLASTP analysis of this sequence did not find any sequences of similar length.

### Biotin Attachment Protein/Pyruvate Carboxylase B

Gene *cfiA* encodes a 597 amino acid product, which was initially annotated as 2-oxoglutarate carboxylase. Subsequent BLASTP searches identified 99% amino acid sequence identity with many *C. coli* biotin attachment proteins. The sequence also shares identity with several conserved domains, including phosphate binding superfamily, carboxylase, carboxylase interaction sites and a biotinylation site. The sequence of aerotolerant *C. coli* OR12 has a T insertion at position 837,990. This results in a frame shift and premature termination at residue 419. BLASTP analysis of the mutant sequence identified no similar sequences of identical length, and lacked part of the carboxylase domain as well as the entirety of the biotinylation and carboxylase interaction sites.

### PseD/Motility Accessory Factor

Nucleotide position 455,044 lies within a poly-C tract. In wild type *C. coli* OR12 this tract is composed of 10 Cs in 83% of the sequence reads, and 9 Cs in 7% of the sequences. The aerotolerant *C. coli* OR12 sequence has become fixed with 100% 9 Cs at this location. This position is within genes ATE51_00982 and ATE51_00984. In the 10 C configuration, the reading frame ATE51_00982 encodes a 593 amino acid protein that shares high sequence identity with PseD proteins (97 to 100% identity) from many other *C. coli* and *C. jejuni* data base entries. The sequence also has 90% coverage and 71% identity with the sequence of PseD from *C. jejuni* NCTC 11168, which is involved in flagellar modification (McNally et al., 2006). The 9 C configuration, present in the minority of the wild type and all of the aerotolerant *C. coli* OR12 sequences, results in a frame shift at the fifth amino acid residue and protein termination at 21 amino acids. The 10 C configuration of gene ATE51_00984 encodes a 60 amino acid protein. However, the 9 C configuration results in a frame shift after 60 amino acid residues and fusion with ATE51_00982 resulting in a 648 amino acid protein, which has 99% coverage and 72% identity with the PseD from *C. jejuni* NCTC 11168.

### Filamentous Haemagglutinin

Gene *hxuA* encodes a 1063 amino acid protein that was annotated as a heme/hemopexin binding precursor. However, BLASTP analysis of the protein sequence identified many *C. coli* filamentous haemagglutinin proteins with over 90% coverage and identity. The amino acid sequence also contains a conserved *N*-terminal haemagglutinin domain, conserved in many proteins with haemagglutination or haemolysin activity. Nucleotide 565,851 lies within a poly-G tract. Of the wild-type *C. coli* OR12 sequences, 96% of contained 10 Gs and 4% contained 9 Gs. Aerotolerant *C. coli* OR12 sequences were composed of 75% containing 9Gs and 25% containing 10 Gs. The 10 G configuration results in encoding of the full 1063 amino acid protein; however, the 9 G configuration results in a frame shift at residue 19 and immediate termination.

### PepD

Gene *pepD* is a disrupted pseudogene in the majority of *C. coli* OR12 sequences. Nucleotide 828,036 lies within a poly-C tract. The wild-type *C. coli* OR12 sequences are composed of: 91% containing 8 Cs and 9% containing 9 Gs. Aerotolerant *C. coli* OR12 sequences are composed of: 77% containing 10 Gs and 23% containing 9 Gs. The 8 G configuration, which represents the majority of wild-type sequences, encodes a 221 amino acid protein that shares high sequence identity (83% coverage and 100% identity) with many *C. coli* aminoacyl-histidine dipeptidase proteins that are significantly longer. The 9 C configuration, present in the minority of wild-type and aerotolerant sequences, encodes a 423 amino acid protein, which has high sequence identity (99%) with other *C. coli* aminoacyl-histidine dipeptidase sequences of the same length. The sequence also shares similarity with a conserved zinc peptidase like superfamily domain. The 10 C configuration, present in the minority of aerotolerant sequences, encodes a 194 amino acid protein, which is identical to the 8 G product apart from the final 9 amino acid residues. Of the protein sequences that show high similarity with the 8 G and 10 G products, none ended in the same location or with similar C-terminal amino acid residues.

### Carbonic Anhydrases

The first 192 amino acids of this predicted 205 amino acid protein product of gene ATE51_00954 share 67% identity with the first portion of the adjacent ATE51_00952 and nearby ATE51_00958, both of which are annotated as putative carbonic anhydrases. BLASTP analysis indicates 100% identity with several 196 amino acid proteins in *C. coli* and *C. jejuni* also similarly annotated as carbonic anhydrases. Aerotolerant *C. coli* OR12 has a single G homopolymer deletion at position 483,767 which causes a frame shift at the G196 residue, replacing residues GGHMEDTYLYS with GVIWRIPIFTHSKDSSLVCGWRD at the carboxyl terminus. BLASTP analysis of the 217 aa mutant configuration identified 88–90% identity with one *C. jejuni* and one *C. coli* hypothetical protein of 218 aa length, however, the majority of entries demonstrating sequence similarity were for 403–404 aa carbonic anhydrases.

Gene ATE51_00958 encodes a 405 aa protein, annotated as carbonic anhydrase with 97–100% identity with *C. jejuni* and *C. coli* carbonic anhydrase proteins of identical length. Aerotolerant *C. coli* OR12 has a G deletion at nucleotide 484,990 which induces a frame shift at residue 197, altering the aa sequence with a stop at 201 aa. BLASTP analysis of the mutant sequence revealed 97% identity with two *C. coli* hypothetical proteins of identical length, one of which was in *C. coli* RM2228.

Nucleotide position 1,158,035 lies within a homopolymeric tract, with wild-type *C. coli* OR12 sequences consisting of: 90% with 10 Cs; 7% with 9 Cs and 3% with 11 Cs. Aerotolerant *C. coli* OR12 sequences consist of: 85% with 8 Cs and 15% with 9 Cs. The original annotation of ATE51_02354 indicated a hypothetical protein; however, a more recent annotation of the *C. coli* OR12 genome within the Genbank database classified the region as a disrupted carbonic anhydrase pseudogene. The 10 C configuration, representing 90% of wild-type *C. coli* OR12 sequences, results in a predicted 190 aa hypothetical protein, with many highly similar hypothetical proteins in the database. One entry suggests high sequence identity with a partial carbonic anhydrase in *C. coli* (entry KQH53282.1). The 8 C configuration, representing 85% of aerotolerant *C. coli* OR12 sequence reads, results in a frame shift and exchange of the terminal GY residue for VLERFCLEIHMADI. This arrangement is also present in several similar sequences in the database, again mostly annotated as hypothetical proteins. The 9 C configuration, present in 7% of wild type and 15% of aerotolerant *C. coli* OR12 sequence reads results in a frame shift and fusion with the next open reading frame, ATE51_02352, resulting in a 408 amino acid protein. This sequence is also present in the database, with all similar entries annotated as hypothetical proteins. Residues 194–408 have a 99% similarity to a putative carbonic anhydrase domain in *C. jejuni* DFVF1099 (Takamiya et al., 2011). The 11 C configuration (3% of wild-type *C. coli* OR12 sequence reads) results in a frame shift similar to the 8 C configuration, but an additional G residue is retained; only the terminal Y amino acid residue is exchanged for the residues VLERFCLEIHMADI. This configuration is less frequently reported in the database than that of the 8C variant. All variants result in high similarity with the conserved domain DUF2920, a bacterial domain of unknown function. The three putative carbonic anhydrase protein sequences above share significant identity at their N-termini, as demonstrated by the alignment in Supplementary Figure 5.

### Oxidative Stress Resistance Genes

Genes involved in *Campylobacter* oxidative stress resistance, as cataloged in a recent review by Flint et al. (2016b), were identified in *C. coli* OR12 and the corresponding protein sequences compared with orthologs from *C. jejuni* NCTC11168. These data are presented in Supplementary Table 1. All the functional genes cataloged by Flint et al. (2016b) could be identified with the exception of those encoding the regulators of response to peroxide A and B (*rrpA* and *rrpB*). No significant sequence similarity to the latter two genes could be identified in the sequences of *C. coli* OR12 or *C. coli* RM1875 or *C. coli* 15-537360.

## Discussion

Aerotolerance of *Campylobacter* spp. may represent a survival adaptation, potentially allowing superior environmental persistence and therefore improved opportunities for colonization of new hosts. The ability to withstand atmospheric oxygen is also relevant to survival on chicken meat and is therefore of public health significance. Aerotolerance is related, at least in part, to oxidative stress resistance which has been shown to be a colonization factor for chickens and a virulence factor for human infection (Hermans et al., 2011; Bolton, 2015; Flint et al., 2016a).

Aerotolerance following aerobic passage of *C. coli* OR12 was demonstrated by aerobic growth on both BA and CCDA. Similar viable counts of wild type *C. coli* OR12 and *C. coli* RM2228 were inoculated to BA, but they failed to grow and had declined below limit of detection by 24 h. Aerobic growth of *C. coli* OR12 Aer was noted at 37°C but not at 42°C. Microaerobic passage did not affect the ability of the *C. coli* OR12 Aer to grow aerobically, indicating that the aerotolerant phenotype is stable and likely due to genetic changes rather than temporary physiological adaptation. No difference in swarming motility was observed between the wild type and aerotolerant *C. coli* OR12. Further evidence of motility was provided by a membrane filtration assay, where growth occurred on BA following inoculation through a 0.45 μm nitrocellulose filter. Impaired motility has been reported to significantly reduce the ability of campylobacters to pass through cellulose membranes (Speegle et al., 2009). This suggests that the aerobic growth can be initiated by motile cells and is not solely facilitated by a protective layer of non-viable cells, shielding viable cells from atmospheric oxygen. Aerotolerant *C. coli* OR12 demonstrated superior resistance to H_2_O_2_ than either wild type *C. coli* OR12 or *C. coli* RM2228. However, *C. coli* OR12 Aer pre-cultured under microaerobic conditions was more sensitive to H_2_O_2_ than an inoculum from an aerobic culture. Aerobic growth may therefore induce factors which render the cells less sensitive to peroxide stress.

Transmission electron microscopy was performed to determine whether the aerotolerant adaptation was accompanied by any changes in cell morphology, for example cell filamentation was noted by Ghaffar et al. (2015) upon entry to stationary phase in broth cultures. No differences in cell morphology were apparent between the wild type and aerotolerant *C. coli* OR12, with spiral, filamentous and coccid cells present within both populations.

Pulsed-field gel electrophoresis of four *C. coli* OR12 Aer isolates, which had been aerobically passaged in parallel, showed no difference to the parent strain. As well as confirming that the isolates were all *C. coli* OR12 and had not been contaminated, this suggested that the aerotolerance was not associated with any major recombination events, such as the genomic rearrangements observed in *C. jejuni* HPC5 in response to phage predation reported by Scott et al. (2007). The changes associated with aerotolerance were therefore considered more likely to be due to point mutations, and thus, whole genome sequencing was performed to investigate this.

### Previous Reports of Aerotolerance

Several authors have reported aerotolerance of *C. jejuni* and *C. coli*, however, the methods adopted to assess this are variable. For example, Jones et al. (1993) report aerobic growth of *C. jejuni* strains on blood agar after 2–3 days of aerobic incubation, but used an initial acclimation period of 18 h under microaerobic conditions. Similarly, Chynoweth et al. (1998) found 29 of 40 *C. jejuni* isolates from various sources could be adapted to grow aerobically on nutrient agar. All isolates were grown microaerobically before streaking on nutrient agar, and though some were initially slow to grow, they became adapted following aerobic subculture. Gaynor et al. (2004) reported the aerobic passage of *C. jejuni* NCTC 11168 on blood agar with antibiotic supplement at 37°C, which resulted in significant attenuation of colonization in a day-old chick model after thirteen passages.

In contrast, Oh et al. (2015) examined aerotolerance of 70 *C. jejuni* isolates obtained from chicken meat samples with respect to survival of approximately 10^9^ CFU/ml in aerobic conditions at 42°C in MHB. Of these, 50 were considered aerotolerant based on survival for 12 h and 25 classified as hyper-aerotolerant, with survival for 24 h. The importance of alkyl hydroperoxide reductase was demonstrated by mutation of *ahpC* leading to the loss of hyper aerotolerance (Oh et al., 2015). Purdy et al. (1999) studied the effect of *sodB* mutation on aerotolerance and *in vivo* colonization fitness of *C. coli*. Inactivation of sodB activity did not affect survival of cultures aerobically incubated at 25°C in MHB, however, the colonization of day old chicks was reduced (Purdy et al., 1999). The aerotolerance test used by Purdy may not be equivalent to that described by Oh et al. (2015), as oxidative stress has been demonstrated to have a significantly greater effect at 42°C that at 25°C (Garénaux et al., 2008).

Kaakoush et al. (2007) examined the growth at several cell densities of four *C. jejuni* strains from aerobically and microaerobically incubated brain heart infusion broth. High initial bacterial density cultures grew when incubated either aerobically or microaerobically; however, cultures with less than 10^4^ CFU/ml only grew under microaerobic conditions. A combination of lower oxygen saturation in liquid media at 42°C and dense cultures that have a greater oxygen demand will lower the oxygen tension and allow growth. No growth occurred when *C. coli* OR12 Aer or WT were aerobically incubated at 37°C in MHB; indeed similar decline rates were observed for the *C. coli* OR12 derivatives and *C. coli* RM2228.

Rodrigues et al. (2015) recently reported the aerobic growth of *C. jejuni* strain Bf on Karmali agar. For this strain visible growth was reported within 24 h of aerobic incubation at 42°C. Similar to *C. coli* OR12 Aer, growth in liquid medium could not be elicited, and a quantitative assay was used to measure growth on solid medium. Superior resistance to peroxide stress was also noted compared to *C. jejuni* NCTC 11168, which had failed to grow aerobically (Rodrigues et al., 2015). Growth on solid medium creates oxygen gradients, such that facultative anaerobes have been found to be more active on the bottom layer and aerobes more rapidly dividing on the top layer of colonies (Reyrolle and Letellier, 1979). Oxygen gradients in bacterial colonies and solid media have also been measured analytically, with the top 30 μm of a gelatine medium being considered aerobic (Jeanson et al., 2015). Therefore, once initial growth of *Campylobacter* has commenced on a solid medium, bacteria that are not directly exposed to the outside atmosphere may reside in an atmosphere more similar to microaerobic conditions.

### Chicken Colonization

Chicken colonization studies confirmed that wild type *C. coli* OR12 is a robust colonizing strain, with reproducible caecal counts between 10^8^ and 10^9^ CFU/g. However, the aerotolerant strain colonized only 2/7 birds at 3 days and 6/8 birds by 7 days. Birds that were colonized had relatively high counts, with all >7 log_10_ CFU/g. The binary nature of the outcomes suggests that bottlenecking events and stochastic processes are significant factors in the colonization process. They are also suggestive of adaptation occurring between days 3 and 7, where campylobacters, presumably present at levels below the limit of detection, have become acclimated to the intestinal or caecal environment and proliferate to achieve higher colonization levels. These adaptations may not be identical between birds, as suggested by the wider range of caecal counts compared with the original isolate. This experiment further validates the importance of individual caging of chickens during *Campylobacter* colonization assays as advocated by Conlan et al. (2011). Had the birds been group housed, any chickens in which the aerotolerant *C. coli* OR12 had colonized would likely have shed the modified strain in large numbers in feces, potentially rapidly colonizing the other birds in the group. The reduction in initial colonization fitness could have been missed under these circumstances. A reduction in the challenge dose could potentially allow further resolution between groups and may be of value in future studies. Aerotolerant *C. coli* OR12 recovered from chickens were still capable of aerobic growth demonstrating the trait is stable and compatible with chicken colonization, albeit potentially at a lower initial level. However, it is difficult to distinguish the lower levels of colonization from the overall effect of *in vitro* passage, which is known to reduce the colonization fitness (Stern et al., 1988). Nevertheless these findings correlate with the observations of Jones et al. (1993) in that aerotolerant *C. jejuni* recovered from mice retained aerotolerance. The evident adaptation between days 3 and 7 did not lead to any loss of aerotolerance. The ability of *C. coli* OR12 to adapt to aerobic conditions and re-adapt to chicken colonization may be a driver of diversity within the *Campylobacter* population, where the plastic genome allows rapid adaptation to new or stressful conditions. Given the capacity of campylobacters for inter-strain recombination these changes may then be disseminated amongst cohabiting *Campylobacter* populations. The ability to withstand, and grow, in aerobic conditions could be of major importance not only in environmental persistence, allowing fomite transfer between farms, but could also allow prolonged survival on processed chicken carcasses and therefore be of public health importance. Survival of *C. coli* OR12 on chicken skin following refrigeration and freezing was examined by El-Shibiny et al. (2009). Survival rates were comparable with the other *C. jejuni* and *C. coli* strains tested. Similar experiments with aerotolerant *C. coli* OR12 could provide valuable insight as to whether improved aerotolerance *in vitro* translates to superior survival on product, which would be of public health significance. This may be further considered in the context of the recently described aerobic *C. jejuni* strain Bf, which was found to be able to adhere to human epithelial cells and form biofilms under aerobic conditions (Bronnec et al., 2016).

This report describes true aerobic growth, without initial support from microaerobic incubation. With the exception of *C. coli* OR12, no growth was noted from any aerobically incubated *Campylobacter* spp. in this study. However, the equality of growth following aerobic or microaerobic incubation, which had been reported by Jones et al. (1993) was not achieved with *C. coli* OR12. Nevertheless the ability to undergo direct growth in atmospheric oxygen, a trait that was retained post either microaerobic growth or *in vivo* passage through chickens, encouraged us to examine the genetic basis for *C. coli* OR12 aerotolerance.

### Genomic Analysis

Oxidative stress resistance has been proposed as a key feature of aerotolerance. Almost all the genes whose products have been implicated in oxidative stress resistance could be identified in *C. coli* OR12, none contained any mutations in the aerotolerant *C. coli* OR12. Orthologs of *rrpA* and *rrpB*, encoding MarR-like transcription factors implicated in oxidative stress resistance, could not be identified in the genome sequences of *C. coli* OR12 or RM1875 or 15-537360 (Gundogdu et al., 2015). Bronnec et al. (2016) identified a complete type VI secretion system in *C. jejuni* Bf and suggested that it may contribute to the aerotolerant phenotype. The *C. coli* OR12 genome contains a complete T6SS, although *C. coli* RM2228, which did not demonstrate aerotolerance, also possesses a full T6SS (Bleumink-Pluym et al., 2013). This would suggest that possession of T6SS alone does not confer aerotolerance, although it could be involved when in combination with other features. Changes in 23 coding sequences were observed between the wild type and aerotolerant *C. coli* OR12 genomes. The functional significances of these are discussed in the following paragraphs.

### Phosphoglycerate Kinase

**FIGURE 6:**
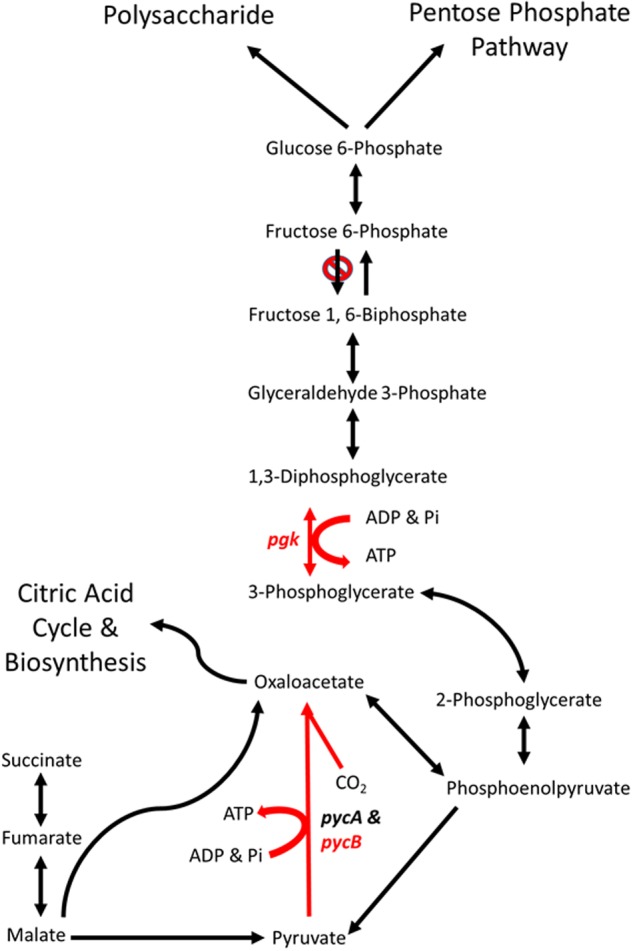
**Schematic representations of the major gluconeogenic and anaplerotic pathways of *Campylobacter*.** Reaction in red are likely to be affected by the mutations in genes *pgk* (phosphoglycerate kinase) and *pycB* (pyruvate carboxylase B subunit) present in *C. coli* OR12 Aer. Modified from Velayudhan and Kelly (2002).

Phosphoglycerate kinase (PGK) is a magnesium dependent kinase involved in glycolysis and gluconeogenesis. In many organisms it performs the first ATP- generating step in glycolysis, however, *C. jejuni* lacks a homolog of 6-phosphofructokinase and therefore cannot metabolize hexose sugars (Velayudhan and Kelly, 2002). Under these circumstances *C. jejuni* PGK is likely to be limited to the gluconeogenic pathway, converting 3-phosphoglycerate to 1,3-diphosphoglycerate (**Figure 6**).

The aerotolerant *C. coli* OR12 sequence has an insertion early in the *pgk* gene that causes a frame shift and termination at 21 amino acids. No other copies of *pgk* are present, so it is likely that no functional PGK protein is produced. This may prevent the gluconeogenic pathway from occurring in aerotolerant *C. coli* OR12, so sugar anabolism must be occurring via different pathways. The *C coli* OR12 strain does possess orthologs to the *serABC* genes present in *C. jejuni* NCTC11168 (ATE51_01764; ATE51_03664; ATE51_03558, BLASTP identities of 94, 79, and 87%, respectively). Therefore, serine biosynthesis from 3-phosphoglycerate should be unaffected (Velayudhan et al., 2004). Circumvention of PGK and reinstatement of gluconeogenesis is theoretically possible via the formation of 2,3-bisphospho-D-glycerate, which would require the enzyme bisphosphoglycerate mutase. A putative phosphoglycerate/bisphosphoglycerate mutase has been annotated for *C. jejuni* NCTC11168, for which an ortholog is present in *C. coli* OR12 (ATE51_01624). The accumulation of 3-phosphoglycerate in the absence of PGK could drive this alternative reaction series. Aerobic growth of *C. coli* OR12 on BA plates prompted this study, and it is worth noting that erythrocytes are estimated to contain 4 to 5 mM 2,3-bisphospho-D-glycerate formed by the Luebering-Rapoport glycolytic shunt, which is used to promote the release of oxygen from deoxygenated hemoglobin at times of need by binding the T-state as an allosteric effector (Benesch and Benesch, 1967). The availability of 2,3-bisphospho-D-glycerate, albeit at declining concentrations, could be utilized by campylobacters to reduce or remove the requirement of PGK in gluconeogenesis and increase the affinity of any remaining hemoglobin to bind oxygen. However, the adaptation present in the aerotolerant *C. coli* OR 12 also enables aerobic growth on CCDA. Modifications to central metabolism are emerging amongst campylobacters, for example it was recently reported that 1.7% of thermophilic campylobacters have the potential to metabolize glucose via the Entner-Doudoroff pathway (Vegge et al., 2016). This pathway also permits the metabolism of L-fucose, which has been reported to provide a competitive advantage to *C. jejuni* in a piglet colonization model (Stahl et al., 2011).

### Biotin Attachment Protein

The *cfiA* gene in the wild-type *C. coli* OR12 sequence encodes a putative biotin attachment protein. The protein sequence shares 85% identity with 100% coverage of the pyruvate carboxylase B subunit of *C. jejuni* NCTC 11168. Therefore, this protein is likely to be the ortholog of PycB, encoded by the gene *cj0933c* in *C. jejuni* NCTC 11168. Pyruvate carboxylase in *C. jejuni* is composed of two subunits, PycA and PycB (Velayudhan and Kelly, 2002). These correspond with the biotin carboxylase and biotin carboxyl carrier subunits of a two-subunit type of carboxylase (Goss et al., 1981). The complete enzyme contains biotin and catalyzes the ATP-dependent carboxylation of pyruvate, yielding oxaloacetate. Velayudhan and Kelly (2002) performed mutation studies of *pycB* and *pycA*, and they noted that mutation of *pycB* resulted in significantly reduced growth in BHI broth, and the inability to use lactate, pyruvate or malate as carbon sources.

The *C. coli* OR12 Aer sequence contains a T insertion at nucleotide position 837,990 compared to the wild-type sequence. This would result in a frame shift and premature termination at 419 amino acids. This is likely to affect the activity of the enzyme as it is missing almost one third of its amino acids, including part of the conserved carboxylase domain as well as the entirety of the putative biotinylation and carboxylase interaction sites. This is surprising as the aerotolerant *C. coli* OR12 has a growth rate very similar to the wild type when grown microaerobically in NB2. Colonies on solid medium from microaerobically incubated aerotolerant *C. coli* OR12 were slightly smaller than the wild type, but this is not similar to the *pycB* mutant which had a threefold reduction in growth compared to the wild type (Velayudhan and Kelly, 2002). Comparisons of carbon metabolism in *C. jejuni* and *C. coli* indicate these species share a core set of carbon sources including amino acids, citric acid cycle intermediates and carboxylic acids (Wagley et al., 2014). However, exceptions were noted such as the inability of *C. coli* strains to metabolize D-malic acid and the inability of *C. jejuni* strains to metabolize propionic acid.

### PseD/Motility Accessory Factor

Post-translational glycosylation of flagellar proteins is important for *Campylobacter* virulence. For example, mutation of *pseA* in *C. jejuni* 81-176 resulted in deficiency of flagellar glycosylation with pseudaminic acid and reduced virulence in a ferret model (Guerry et al., 2006). The function of the gene *pseD*, *cj01333* in *C. jejuni* NCTC 11168, which is a member of the motility accessory family, was examined in the context of flagellar glycosylation in *C. jejuni* 81-167 by McNally et al. (2006). Mutation of *pseD* did not affect cell motility or flagellin glycosylation with pseduaminic acid. However, mutation did prevent glycosylation with the pseudaminic acid acetamidino derivative PseAm, though it did not prevent cellular production of PseAm. Changes within a homopoylmeric C tract of *pseD* in aerotolerant *C. coli* OR12 effectively reinstates a longer reading frame to fuse ATE51_00982 and ATE51_00984 to create a 648 amino acid predicted product that exhibits 99% coverage with the *C. jejuni* NCTC 11168 PseD.

### Filamentous Haemagglutinin

The gene *hxuA* encodes a protein which shares high sequence identity with other *C. coli* proteins annotated as filamentous haemagglutinin. The sequence also contains a region with high similarity to several conserved haemagglutination activity domains. Filamentous haemagglutinin proteins are involved in virulence of several bacterial pathogens, including *Bordetella pertussis* and *Avibacterium* (formerly *Haemophilus*) *paragallinarum* (Hobb et al., 2002; Inatsuka et al., 2005). The genome of *Arcobacter butzleri* ATCC 49616 contains the gene *hcpA* which encodes an iron activated filamentous hemagglutinin (Douidah et al., 2012). BLASTP comparison of this protein with *C. coli* OR12 HxuA revealed no significant similarity, nor did comparison with the *C. concisus* HcpA, the HagA protein of *A. paragallinarum*, or the *Bordetella* proteins FhaA and FhaB. *C. fetus* subsp. *fetus* 82-40 encodes a putative filamentous haemagglutinin of the HcpA family. It may be coincidental that this species is associated with bacteraemic infections, however, it is unknown whether this potential virulence factor is functional *in vivo* (Miller, 2008). The protein may have a possible role in iron acquisition or metabolism, which is known to be involved in the oxidative stress response (Atack and Kelly, 2009). The function of the putative filamentous haemagglutinin encoded by *hxuA* in *C. coli* OR12 remains obscure but the reading frame is notable in that it is phase variable, being intact in 96% of the wild-type sequence reads but appears disrupted in aerotolerant *C. coli* OR12.

### Carbonic Anhydrases

Carbonic anhydrase enzymes catalyze the reversible hydration of CO_2_ to bicarbonate and can be divided into at least six different phylogenetic groups, with varying expressions across kingdoms (Bury-Moné et al., 2008; Al-Haideri et al., 2015). These groups have similar function but differing structures, which are believed to have arisen by convergent evolution (Smith and Ferry, 2000). Strains of *C. jejuni* are reported to encode a β and a γ carbonic anhydrase, the former class are oligomeric, containing between 2 and 8 monomers and the latter are homotrimers (Smith and Ferry, 2000). However, the putative carbonic anhydrase annotated genes affected in the aerotolerant *C. coli* OR12 are not similar to those that have been investigated in campylobacters thus far. Whether these represent a different class of carbonic anhydrases not yet characterized in *Campylobacter*, or encode a hitherto unknown function requires further investigation.

Eleven other genes contained coding mutations, all of which involved single nucleotide substitutions or changes of up to three amino acids at the C-terminus. None of these involved genes which could be expected to influence aerotolerance, such as those encoding oxidative stress resistance proteins or their regulatory genes. Further investigation of these changes would include protein modeling software to determine whether the changes induce significant structural alterations or cause relocation of the protein.

## Conclusion

The *C. coli* strain OR12, which is a robust colonizer of chickens, was found to be capable of aerobic growth on blood agar. This aerotolerance became further evident after serial passage and was associated with increased peroxide stress resistance but did not influence cellular morphology or motility. Colonization of chickens by aerotolerant *C. coli* OR12 was lower than the wild-type strain at 3 days after challenge but not by 7 days, suggesting adaptation was occurring. Whole genome sequencing did not reveal any changes in genes known to be related to oxidative stress. Instead, significant mutations were present within genes encoding phosphoglycerate kinase and pyruvate carboxylase, suggesting alterations to cellular metabolism.

## Author Contributions

PO’K and IC designed and executed experiments, analyzed data, prepared and reviewed the manuscript.

## Conflict of Interest Statement

The authors declare that the research was conducted in the absence of any commercial or financial relationships that could be construed as a potential conflict of interest.

## Footnotes

1 https://blast.ncbi.nlm.nih.gov/Blast.cgi
