# Supplementary material for: Characterisation of Aerotolerant Forms of a Robust Chicken Colonizing Campylobacter coli
Source: Front Microbiol. 2017 Mar 27;8:513. doi: 10.3389/fmicb.2017.00513 (PMC5366326; doi:10.3389/fmicb.2017.00513)
Supplement: Supplementary file 1 [file Table_1.DOCX]

**Supplementary Table 1 Genes involved in *Campylobacter* oxidative stress defence.**

Orthologues identified in *C. coli* OR12 and protein sequence similarity with *C. jejuni* NCTC 11168.

|  |  | **Gene in *C. jejuni*** | **Orthologue in** | **Protein BLAST** | |
| --- | --- | --- | --- | --- | --- |
|  |  | **NCTC 11168** | ***C. coli* OR12** | **% Cover** | **% ID** |
| **Oxidative response** |  |  |  |  |  |
| Superoxide dismutase | SodB | *cj0169* | ATE51_03994 | 100 | 98 |
| Catalase | KatA | *cj1385* | ATE51_00790 | 100 | 95 |
| Catalase biogenesis protein | Cj1386 | *cj1386* | ATE51_00788 | 99 | 88 |
| Alkyl hydroperoxide reductase | AhpC | *cj0334* | ATE51_03544 | 100 | 97 |
| Thiol peroxidase | Tpx | *cj0779* | ATE51_02088 | 100 | 93 |
| Bacterioferritn comigratory protein | BCP | *cj0271* | ATE51_03702 | 98 | 91 |
| DNA-binding protein | Dps | *cj1534c* | ATE51_00506 | 100 | 89 |
| Desulforuberythrin | DRbr | *cj0012c* | ATE51_00028 | 100 | 97 |
| Methionine sulfoxide reductase | MsrA | *cj0637c* | ATE51_02318 | 99 | 71 |
| Methionine sulfoxide reductase | MsrB | *cj1112c* | ATE51_01376 | 97 | 91 |
|  |  |  |  |  |  |
| **Regulators** |  |  |  |  |  |
| Peroxide regulator | PerR | *cj0322* | ATE51_03572 | 100 | 84 |
| Ferric uptake regulator | Fur | *cj0400* | ATE51_03414 | 100 | 97 |
| *Campylobacter* oxidative stress regulator | CosR | *cj0355c* | ATE51_03502 | 100 | 98 |
| LysR-trpe regulator | Cj1000 | *cj1000* | ATE51_01628 | 99 | 80 |
| Regulator of response to peroxide | RrpA | *cj1546* | Not identified | N/A | N/A |
| Regulator of response to peroxide | RrpB | *cj1556* | Not identified | N/A | N/A |
