# Supplementary material for: Characterisation of Aerotolerant Forms of a Robust Chicken Colonizing Campylobacter coli
Source: Front Microbiol. 2017 Mar 27;8:513. doi: 10.3389/fmicb.2017.00513 (PMC5366326; doi:10.3389/fmicb.2017.00513)
Supplement: Supplementary file 2 [file Image_1.PDF]

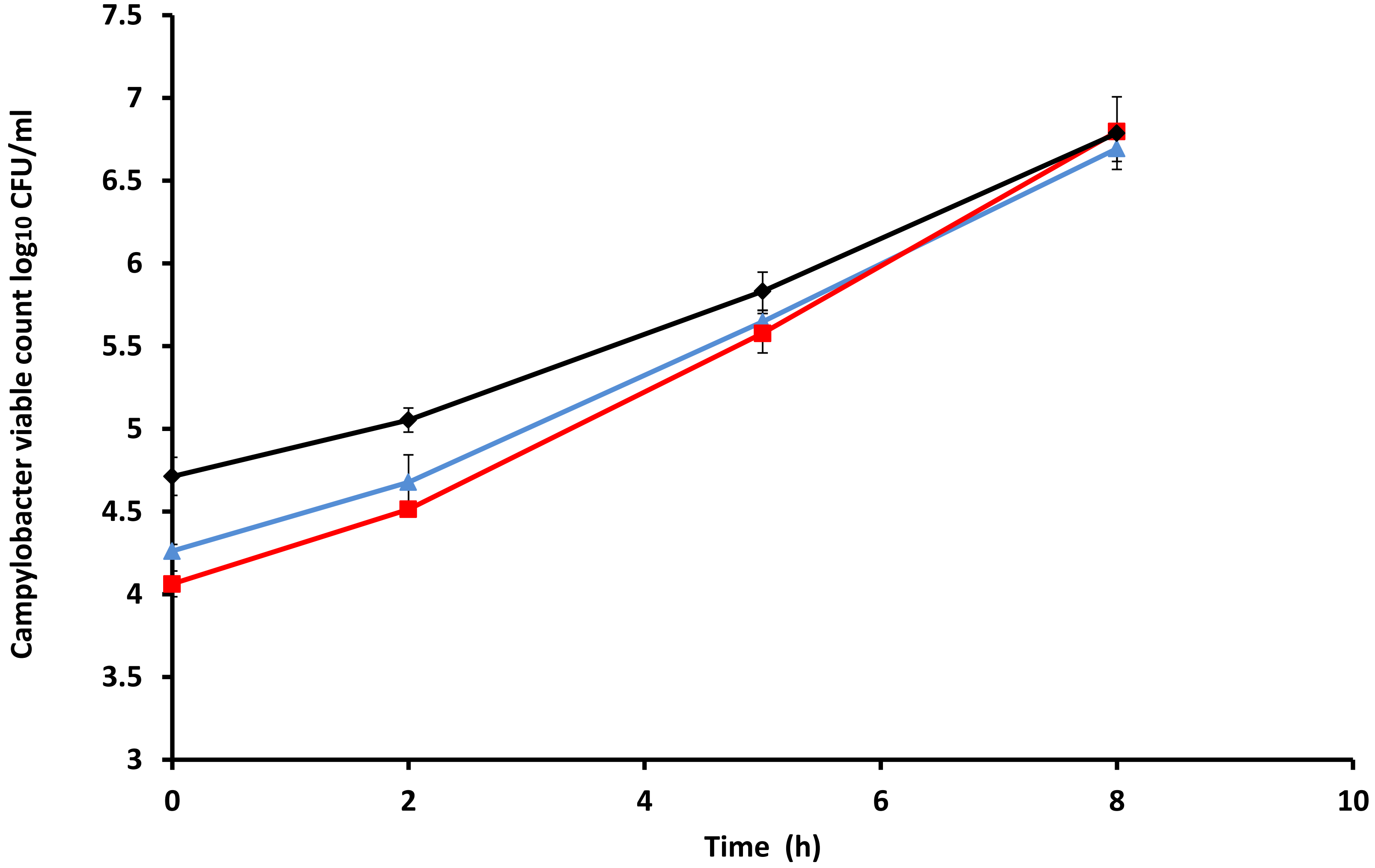

Supplementary Figure 1 Growth of *C. coli* RM2228, OR12 WT and OR12 Aer P38 in microaerobic conditions.

C. coli RM2228 (blue), *C. coli* OR12 WT (red), *C. coli* OR12 Aer P38 (black). Cultures were microaerobically incubated at 42°C in 30 ml of Nutrient Broth No2 with shaking. Error bars represent standard deviations generated from triplicate biological replicates.
