# Supplementary material for: Characterisation of Aerotolerant Forms of a Robust Chicken Colonizing Campylobacter coli
Source: Front Microbiol. 2017 Mar 27;8:513. doi: 10.3389/fmicb.2017.00513 (PMC5366326; doi:10.3389/fmicb.2017.00513)
Supplement: Supplementary file 3 [file Image_2.PDF]

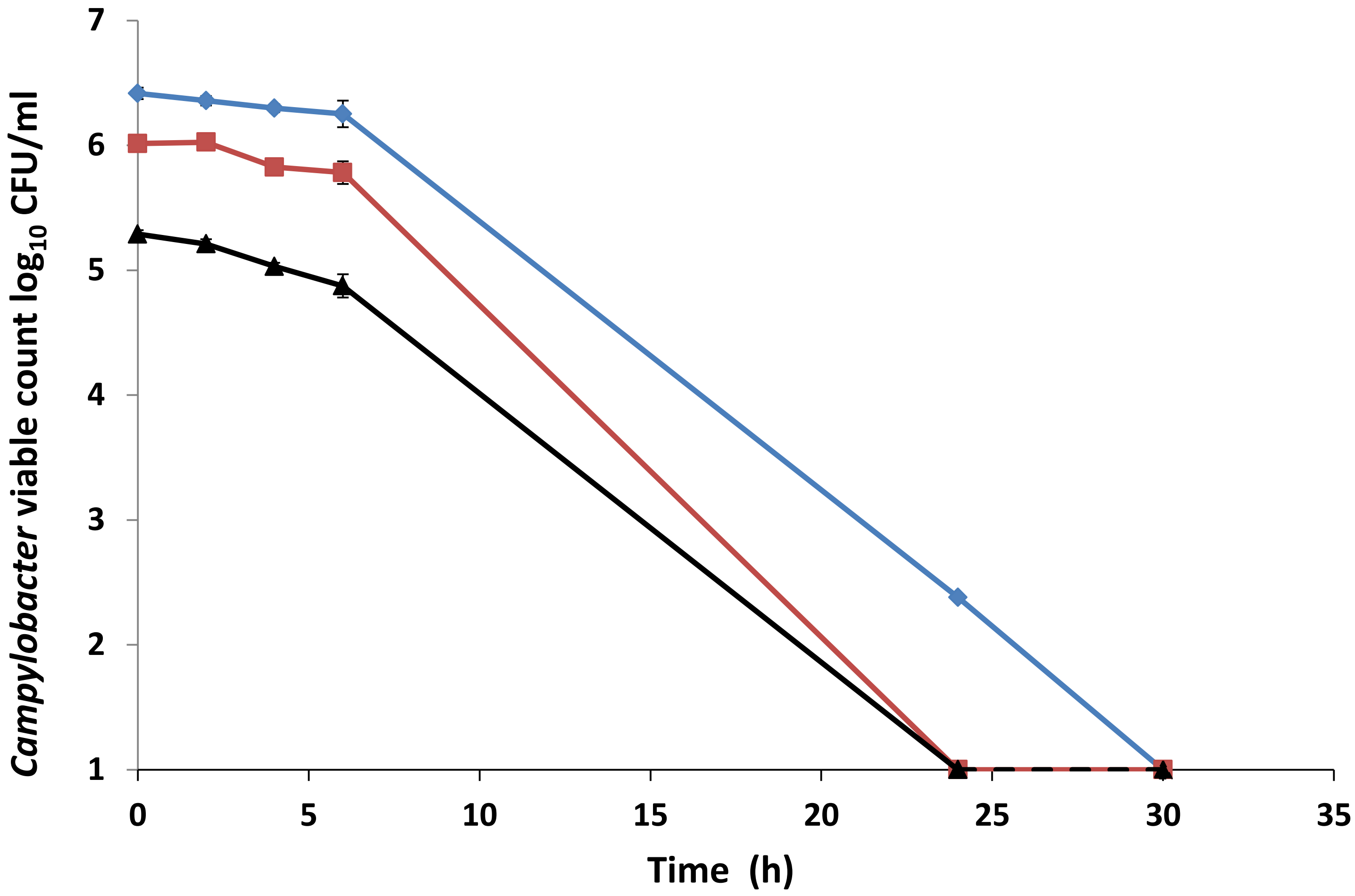

Supplementary Figure 2 Survival of *C. jejuni* HPC5, *C. coli* OR12 WT and *C. coli* OR12 Aer following aerobic incubation at 37°C in MHB.

C. jejuni HPC5 (blue), *C. coli* OR12 WT (red), *C. coli* OR12 Aer P25 (black). Error bars represent standard deviations generated by triplicate biological repeats. Limit of detection was 1.3 $\log_{10}$ CFU/ml.
