# Supplementary material for: Characterisation of Aerotolerant Forms of a Robust Chicken Colonizing Campylobacter coli
Source: Front Microbiol. 2017 Mar 27;8:513. doi: 10.3389/fmicb.2017.00513 (PMC5366326; doi:10.3389/fmicb.2017.00513)
Supplement: Supplementary file 4 [file Image_3.PDF]

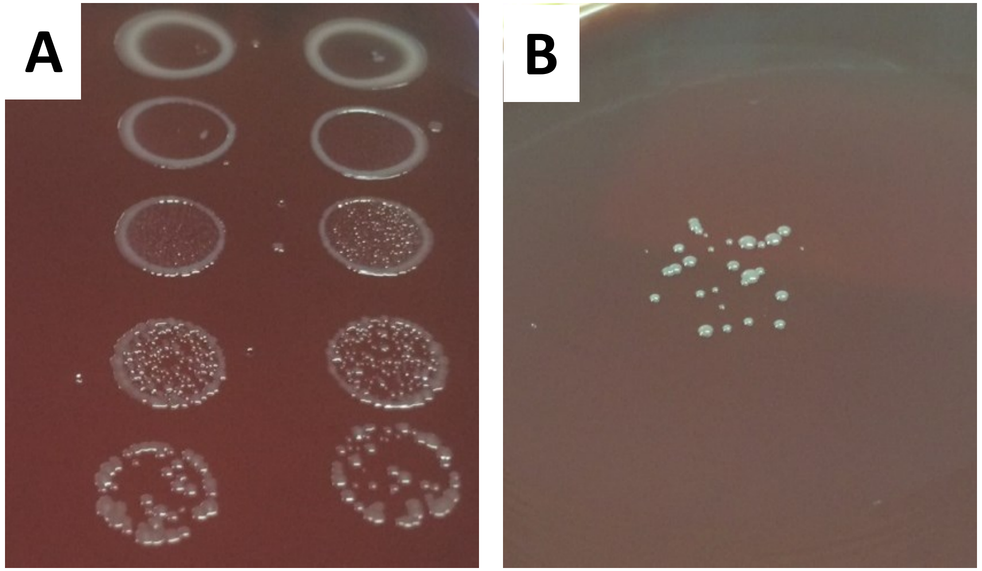

Supplementary figure 3: Aerobic growth of *C. coli* on BA following recovery from chickens challenged with *C. coli* OR12 Aer.

A) Secondary aerotolerance assay, with serially diluted *C. coli* cultured from an initial microaerobic membrane filtration assay, following 5 days of aerobic incubation. B) Direct membrane filtration of cecal content on to a BA plate and incubation for 6 days at 37°C in ambient atmospheric conditions.
