# Supplementary material for: Characterisation of Aerotolerant Forms of a Robust Chicken Colonizing Campylobacter coli
Source: Front Microbiol. 2017 Mar 27;8:513. doi: 10.3389/fmicb.2017.00513 (PMC5366326; doi:10.3389/fmicb.2017.00513)
Supplement: Supplementary file 5 [file Image_4.PDF]

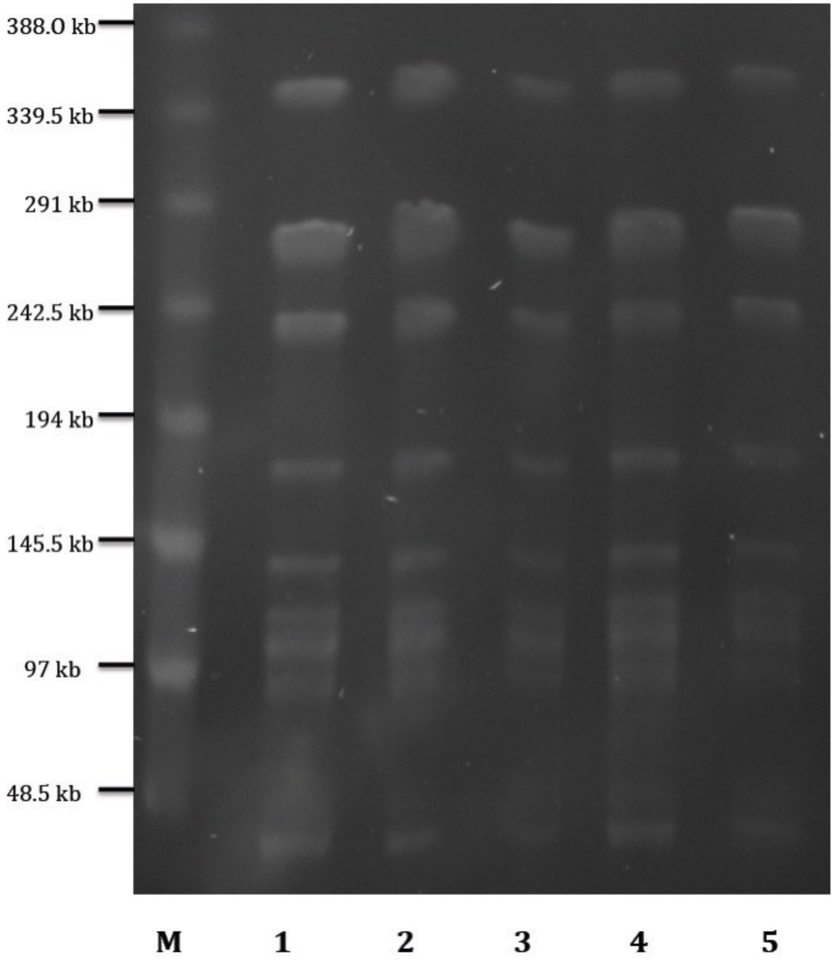

Supplementary Figure 4 PFGE profile of *Sma*I digested genomic DNA of wild type *C. coli* OR12 and four aerobically passaged isolates.

M) Lambda ladder DNA marker; 1) Wild type *C. coli.* OR12; 2-5) four independent aerobically passaged *C. coli.* OR12 isolates (P26).
