# Supplementary material for: Characterisation of Aerotolerant Forms of a Robust Chicken Colonizing Campylobacter coli
Source: Front Microbiol. 2017 Mar 27;8:513. doi: 10.3389/fmicb.2017.00513 (PMC5366326; doi:10.3389/fmicb.2017.00513)
Supplement: Supplementary file 6 [file Image_5.PDF]

```
ATE51_00954_Wild_type          MLINQTFEIDSCDDVELGIKRTSKLEYRISYDDEKEIKAIVFIIGGYGAN
ATE51_00958_Wild_type          MLINQTFEIDSCDDVELGIKRTSKLEYRISYDDEKEIKAIVFIIGGYGAN
ATE51_02354_ATE51_02352_fusion MIINQIYSIDSCDDVELNIKRGSKLEFRLTYDDSKEIEAIVCIIPGGAED
                               *:*** :.*********.*** ****:*::***.***:*** ** * . :

ATE51_00954_Wild_type          ANIYFLDSYRNYIAKNFDVVAVHVFYHCFCARQSIDQKYNPKLIPNKDDL
ATE51_00958_Wild_type          ANIYFLDSYRNYIAKNFDVVAVHVFYHCFCQRRSDVEKYSAYKYFQEEDI
ATE51_02354_ATE51_02352_fusion MNNYIYVDD--YLARNYNVAIININYHCIGNRPHLGSSFYLDDIDKIILD
                                * *:  .   *:*:*::*. ::: ***:  *    ..:      :

ATE51_00954_Wild_type          ERINNILKNINLGHLLANEDNFEQIIPFIEQRAGEIKQAGLVDESQKIGL
ATE51_00958_Wild_type          ENIKNLLNQFHFSYGEINNDNALFLANSLVKHVENLKMQNKLDHNFKLNF
ATE51_02354_ATE51_02352_fusion TSLKTINLNHINVFDINSYENLNNAFIRIDQEIQKLKLNQKLNQNYKLRT
                                 ::.:  :    .   . :*       : :.  ::*    ::.. *:

ATE51_00954_Wild_type          SCDFIPPNGDYQNFGIMAAIDHINALKDLVKRFPKLAD---LPKIYGGGH
ATE51_00958_Wild_type          TSTFISPNGDYQNFGIMAALDHINALKDLVKCFPKFAD---LPKIYGGGS
ATE51_02354_ATE51_02352_fusion HVSFLPSKNEYQNFGIMQAMDILNAIFYIKENSPFKLMGGGIRTILFGNS
                                  *:..:.:******* *:* :**:  : :  *       : .*  *.

ATE51_00954_Wild_type          MEDTYLYS------------------------------------------
ATE51_00958_Wild_type          YGGYLALLIAKIAPWYVDGVIDNSGSALPP--LNYILGREMEHSYGDYY-
ATE51_02354_ATE51_02352_fusion YGGYLANLCAKIAPWSIDFILDNSSFVNLFGNIFRLIGFGKEIDFTRYHG
                                      .

ATE51_00954_Wild_type          --------------------------------------------------
ATE51_00958_Wild_type          ----EDFPHNRIIFFLKTHWTRKENSPYFFNNENYFIRTLLNKDHLILQS
ATE51_02354_ATE51_02352_fusion TYDDTLFKNIFLYLSDKTYWNNNKFSKKYFSNARKIIREPLNKEHLIIQS


ATE51_00954_Wild_type          --------------------------------------------------
ATE51_00958_Wild_type          QKNKNIIYVSYHSKEDPLTPANFKEQTMQILKILGYDVSLNLIDENKIDG
ATE51_02354_ATE51_02352_fusion LYP-NPKYILYHSIFDERSPFENKENFVHILKELNFKVEFFAVS--QVDN


ATE51_00954_Wild_type          --------------------------------------------------
ATE51_00958_Wild_type          KFIKNLDHGCGIPDKALFRKELPLMLEKLQGRKSFMQENSISYPCGNKVF
ATE51_02354_ATE51_02352_fusion KFIKNLNHGMGLSTKLFFKKHLLQILKEP--LQDKICKKEVSYKCDELVY


ATE51_00954_Wild_type          ---------------
ATE51_00958_Wild_type          TFKDVGDKFELEIKD
ATE51_02354_ATE51_02352_fusion TFKEENHQIILNITN
```

Supplementary Figure 5: Alignment of three putative carbonic anhydrase protein sequences.
The longest variant of each protein was selected: wild type *C. coli* OR12 ATE51_00954 and ATE51_00958, and the fused ATE51_02354 & ATE51_02352 variant in the minority of wild type and aerotolerant *C. coli* OR12 sequences. Performed using multiple sequence alignment tool CLUSTALW (http://www.genome.jp/tools/clustalw/).
